# *In silico* analysis of pathways activation landscape in oral squamous cell carcinoma and oral leukoplakia

**DOI:** 10.1038/cddiscovery.2017.22

**Published:** 2017-05-22

**Authors:** Eugene Makarev, Adrian D Schubert, Riya R Kanherkar, Nyall London, Mahder Teka, Ivan Ozerov, Ksenia Lezhnina, Atul Bedi, Rajani Ravi, Rannee Mehra, Mohammad O Hoque, Ido Sloma, Daria A Gaykalova, Antonei B Csoka, David Sidransky, Alex Zhavoronkov, Evgeny Izumchenko

**Affiliations:** 1 Insilico Medicine, Inc., Emerging Technology Centers, Johns Hopkins University at Eastern, B301, 1101 33rd Street, Baltimore, MD 21218, USA; 2 Department of Otolaryngology-Head and Neck Cancer Research, Johns Hopkins University, School of Medicine, Baltimore, MD, USA; 3 Department of Anatomy, Howard University, Washington, DC, USA; 4 Department of Oncology, Johns Hopkins University, School of Medicine, Baltimore, MD, USA; 5 R&D, Champions Oncology, Baltimore, MD, USA; 6 D. Rogachev Federal Research and Clinical Center for Pediatric Hematology, Oncology, and Immunology, Samory Mashela 1, Moscow 117997, Russia; 7 The Biogerontology Research Foundation, 2354 Chynoweth House, Trevissome Park, Truro TR4 8UN, UK

## Abstract

A subset of patients with oral squamous cell carcinoma (OSCC), the most common subtype of head and neck squamous cell carcinoma (HNSCC), harbor dysplastic lesions (often visually identified as leukoplakia) prior to cancer diagnosis. Although evidence suggest that leukoplakia represents an initial step in the progression to cancer, signaling networks driving this progression are poorly understood. Here, we applied *in silico* Pathway Activation Network Decomposition Analysis (iPANDA), a new bioinformatics software suite for qualitative analysis of intracellular signaling pathway activation using transcriptomic data, to assess a network of molecular signaling in OSCC and pre-neoplastic oral lesions. In tumor samples, our analysis detected major conserved mitogenic and survival signaling pathways strongly associated with HNSCC, suggesting that some of the pathways identified by our algorithm, but not yet validated as HNSCC related, may be attractive targets for future research. While pathways activation landscape in the majority of leukoplakias was different from that seen in OSCC, a subset of pre-neoplastic lesions has demonstrated some degree of similarity to the signaling profile seen in tumors, including dysregulation of the cancer-driving pathways related to survival and apoptosis. These results suggest that dysregulation of these signaling networks may be the driving force behind the early stages of OSCC tumorigenesis. While future studies with larger leukoplakia data sets are warranted to further estimate the values of this approach for capturing signaling features that characterize relevant lesions that actually progress to cancers, our platform proposes a promising new approach for detecting cancer-promoting pathways and tailoring the right therapy to prevent tumorigenesis.

## Introduction

Head and neck squamous cell carcinoma (HNSCC) is the sixth most common cancer in the world,^1^ with an annual incidence of over 50 000 cases in the United States alone.^2^ Despite improvements in molecular diagnosis, oral squamous cell carcinoma (OSCC), the most common subtype of HNSCC, has a poor prognosis with a 5-year overall survival of ~50%,^3–5^ and no targeted curative treatments for late-stage OSCC are currently available. As such, there is a significant unmet clinical need for development of novel targeted approaches against OSCC.

Nearly 20% of patients with OSCC harbor multiple pre-malignant lesions showing signs of dysplasia, often visually identified as leukoplakia.^6^ As some of these lesions evolve to malignant neoplasms,^6,7^ they represent intermediate steps in OSCC progression.^8^ It has been postulated, that this multistep process from normal epithelium to early pre-malignant change to fully invasive OSCC, involves the accumulation of molecular and cellular changes.^3,9^ Indeed, several signaling pathways have been shown to be dysregulated in OSCC through genetic and epigenetic alterations such as those involving *TP53*, *PIK3CA*,* NOTCH1*,* SMAD4*, *CDKN2A*, *CCND1*, *FBXW7*, *HRAS*, *NRAS* and *FAT1*.^10–16^ Although these signaling axes have been implicated in tumorigenesis, and are correlated with poor patients prognosis, relatively few molecular changes critical to the progression of oral leukoplakia to OSCC are currently recognized,^17–21^ and our understanding of the complex signaling networks that both promote OSCC pathogenesis and can be targeted therapeutically is still limited.

The Gene Expression Omnibus (GEO), the largest public repository for high-throughput gene expression data, provides unprecedented opportunities to reveal molecular mechanisms of cancer. Although multiple efforts taken to identify differentially expressed genes between OSCC and normal samples have led to the identification of prospective genetic biomarkers and expression signature patterns,^22–27^ analyses that rely solely on gene enrichment statistics fail to capture subtle differences between samples that arise from dynamic interactions between genes at the level of signaling networks. The major advantage of pathway-based methods is their capability to perform biologically relevant dimensionality reduction as a result of the analysis.^28^ However, despite the significant advancements in large-scale analytical methodologies that infer complex transcriptomic changes into the network of biologically relevant signaling axes,^29–33^ a systematic comprehensive analysis of the signaling pathways activation in OSCC and oral pre-neoplastic lesions has never been done.

We have recently introduced the *in silico* Pathway Activation Network Decomposition Analysis (iPANDA) as a scalable robust method for quantitative and qualitative large-scale transcriptomic data analysis.^34^ The iPANDA method combines pre-calculated gene co-expression data with gene importance factors based on the degree of differential gene expression and pathway topology decomposition for obtaining pathway activation scores. It has been demonstrated that iPANDA provides significant noise reduction in transcriptomic data and identifies highly robust sets of biologically relevant pathway signatures.^34^ In this study, we applied iPANDA on the transcriptomic data from hundreds of OSCC and leukoplakia samples to identify the differentially dysregulated signaling pathways between these neoplasms and normal oral mucosa tissues. Our work further contributes to the current understanding of the complex signaling networks underlying HNSCC, and may aid in the development of novel means of prevention, diagnosis and treatment.

## Results

### iPANDA detects major conserved pathways involved in HNSCC tumorigenesis

**Figure 1:**
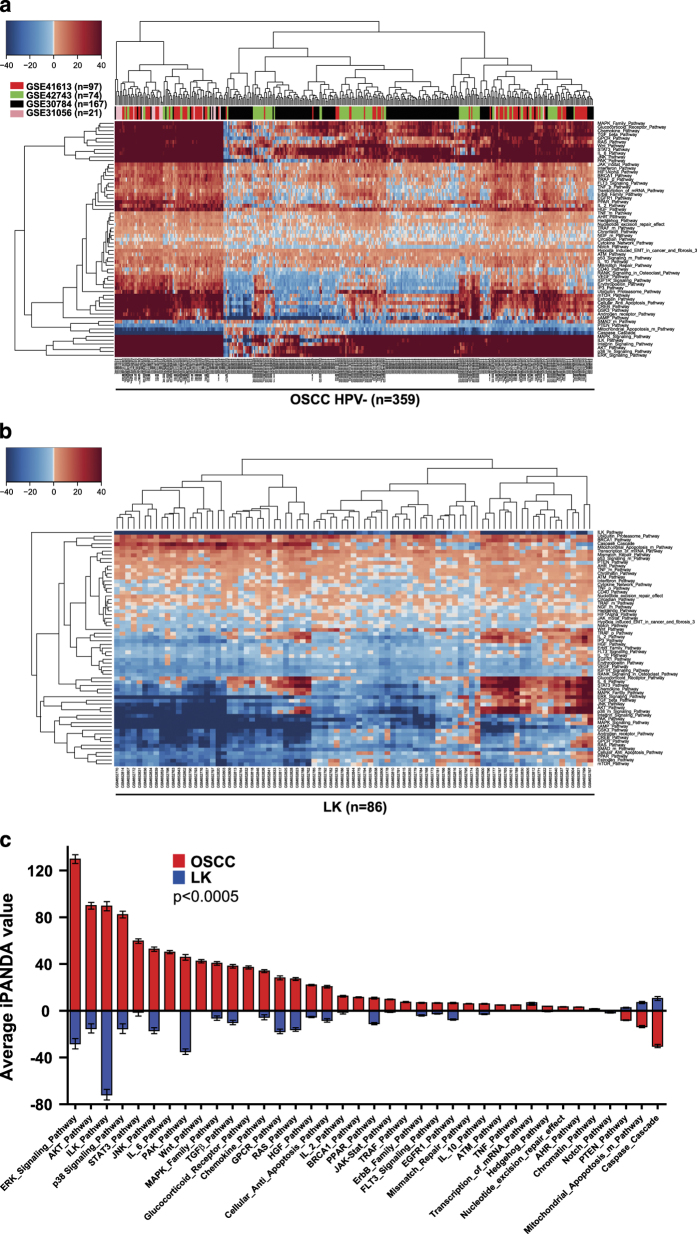
Pathway activation analysis in OSCC and oral dysplastic lesions. (**a**) Transcriptomes from 359 OSCC samples were processed and analyzed using the iPANDA software suite. Transcriptomic data derived from non-tumorous oral mucosa of 109 subjects was used as a reference for analysis. The hierarchically clustered heatmap depicts 64 main signaling pathways dysregulated in OSCC samples. Downregulated iPANDA values for each sample/pathway are indicated in blue, whilst upregulated values are shaded in red. (**b**) Hierarchically clustered heatmap represents 64 main differentially activated pathways in 86 oral dysplasia samples. Transcriptomic data from healthy oral mucosa processes on the same platform was used as a reference. Red boxes represent pathway upregulation and blue boxes represent pathway downregulation. (**c**) Superimposed bar chart depicts the differential expression of 38 main pathways significantly dysregulated (*P<*0.0005) in both OSCC (red bars) and leukoplakia (blue bars) samples.

To assess the signaling pathway profile associated with the OSCC evolution, we first performed an *in silico* pathway activation analysis of 359 OSCC samples obtained from the publicly available data sets deposited at NCBI GEO database (GSE41613, GSE42743, GSE30784 and GSE31056) (Supplementary Table 1). All the data sets were obtained using the microarray platform Affymetrix Human Genome U133 Plus 2.0 Array. Human papillomavirus (HPV) infection does not appear to be a significant risk factor for OSCC and the prevalence of HPV-positive oral cavity cancers is relatively low, at <6%.^35^ To avoid the possible noise introduced by differences in gene expression in HPV-positive and HPV-negative cancers, all OSCC samples selected for the analysis were HPV negative. For OSCC cohort used in this study, we have carefully selected a tissue-specific normal control cohort (microarrays derived from non-tumorous oral mucosa of 109 subjects using the same profiling platform as in tumor data set) (Supplementary Table 2). A collection of 374 intracellular signaling pathways strongly implicated with various solid malignancies (64 main signaling pathways and 310 branched axes radiating from the main pathways) was obtained from the SABiosciences (http://www.sabiosciences.com/pathwaycentral.php), and used for the computational algorithm as described previously.^28,32–34,36^ Using the normal samples cohort as a reference, we determined a quantitative measure of the signaling pathway activation scores (which we refer to as iPANDA values)^34^ for each one of the signaling axes analyzed. The iPANDA values represent the signed scores showing the intensity and direction of the pathway activation (see Materials and Methods section for details). A total of 248 of 374 pathways analyzed (including the 64 main pathways) were significantly dysregulated (*P*<0.05) in OSCC tumors when these samples were compared with non-tumorous oral mucosa (Supplementary Table 3). Focusing on the major pathways involved in cancer development, we have created the hierarchically clustered heatmap of 64 main pathways differentially activated in 359 OSCC tumors (Figure 1a, Supplementary Table 4). Upregulated pathways can be seen in red and downregulated in blue. As expected, pathways involved in initiation, progression and maintenance of HNSCC,^37,38^ such as those associated with AKT, JNK, IL-6/STAT3, ILK, RAS, MAPK/ERK, p38/PAK, TGF*β*, PI3K/mTOR and WNT signaling, were among the most significantly upregulated molecular axes in OSCC patients compared to normal controls (Figure 1a). Our pathway activation prediction analysis further confirms previously reported observations that despite the remarkable complexity of genomic alterations in HNSCC, most of them fall within a few major canonical driver-signaling pathways, and converge the activation of PI3K/AKT/mTOR, RAS/RAF/MAPK, JAK/STAT, WNT/*β*-catenin and TGF*β* molecular networks. Activation of these pathways has been shown to contribute to the malignant growth and metastatic potential of HNSCC.^37–39^

Loss of SMAD4 activity via inactivating mutations or loss of heterozygosity occurs in ~50% of patients with HNSCC, and Smad4 deficiency is associated with elevated TGF*β* expression and spontaneous HNSCC formation.^15^ Whereas activation of PI3K/AKT via loss of *PTEN* or copy-number gain/activating mutations in *PIK3CA* also occurs in 40–60% of HNSCCs, leading to increased IL-6 production, STAT3 activation and tumor-promoting inflammation in HNSCC stroma.^40,41^ In concordance with this evidence, PTEN and SMAD signaling, as well as pathways associated with apoptosis were significantly downregulated in the cohort of 359 OSCC tumors analyzed (Figure 1a).

### Analysis of signaling landscape in pre-malignant leukoplakia lesions

Given the difficulties to distinguish pre-neoplastic oral lesions from cancer, there is an urgent need to elucidate the complexity of signaling processes underlying the progression from leukoplakia to OSCC. Therefore, we next applied iPANDA algorithm to obtain pathway activation scores based on gene expression profiles of 86 oral pre-neoplastic lesions (GSE26549) (Supplementary Table 5). Given the difficulty of obtaining gene expression data of well-annotated leukoplakia specimens, this was the only data set currently available. Since gene expression profiles for these samples were obtained using the later version of the Affymetrix array (1.0 ST Array), as a reference, we have selected a cohort of non-tumorous oral mucosa samples profiled on the same platform (GSE38616) (Supplementary Table 6). Out of the 374 pathways analyzed, 210 pathways (64 main pathways and 146 branches) were significantly dysregulated (*P<0.05*) in leukoplakia samples compared to healthy controls (Supplementary Table 7). The heatmap for hierarchically clustered 64 main pathways differentially activated across the 86 leukoplakia samples is shown in Figure 1b. Interestingly, pathways activation landscape in leukoplakia was profoundly different from that seen in OSCC. To better visualize these differences, we have created a superimposed bar chart, which depicts the differential expression of 38 main pathways significantly dysregulated (*P*<0.0005) in both OSCC (red bars) and leukoplakia (blue bars) samples (Figure 1c). Unlike in OSCC, the average levels of iPANDA values for AKT/mTOR and other survival and mitogenic pathways, such as ERK, JNK, RAS/MAPK, p38, PAK, integrin/ILK and TGF*β* signaling, were substantially lower in most leukoplakia samples when compared to the normal cohort (Figure 1b, Supplementary Table 8). Since it was postulated that only a subset of pre-malignant oral lesions may progress to invasive cancer, these data may point to the possibility that downregulation of molecular pathways associated with proliferation and survival may direct pre-neoplastic lesions to spontaneously regress and not to progress to cancer. Supporting this suggestion, in the majority of the pre-neoplastic samples, pro-apoptotic pathways were predicted to be slightly upregulated compared to the normal controls (Figure 1b, Supplementary Table 8). Notably, similar to OSCC, the SMAD signaling pathway was also downregulated in leukoplakia. Since reduced SMAD4 expression may occur in a fairly large percent (~40%) of oral pre-neoplastic lesions,^15,42^ our data further support the role of SMAD4 alterations is an early event in OSCC tumorigenesis.

Many well-established cancer-driving genes simultaneously modulate activation or repression of multifunctional signaling networks that control cell growth, differentiation and migration. Since ‘main’ pathways aggregate branches that may be inversely activated, analysis of the pathway branches (which represent linearized fragments of the main pathway networks with multiple downstream targets) may help to scale down the complexity of common non-linear topology^34,43^ and potentially unravel the most critical branches associated with cancer progression. A list of pathway branches predicted to be significantly dysregulated (*P*<0.0005) in both OSCC and leukoplakia samples is shown in Supplementary Table 9. Notably, several sub-branches of ILK, IGF1R, HIF1, HGF, MAPK and WNT pathways were among the most differentially dysregulated axes between OSCC and leukoplakia (Supplementary Table 9), suggesting their role in the transition from non-invasive to invasive disease. Although analysis of the distinct linear elements within a large multifunctional cancer driver network may provide a more comprehensive signaling signature of the phenotype under investigation, the true value of these subaxes in HNSCC is a subject of future investigation.

### Possible harbingers of malignant transition

**Figure 2:**
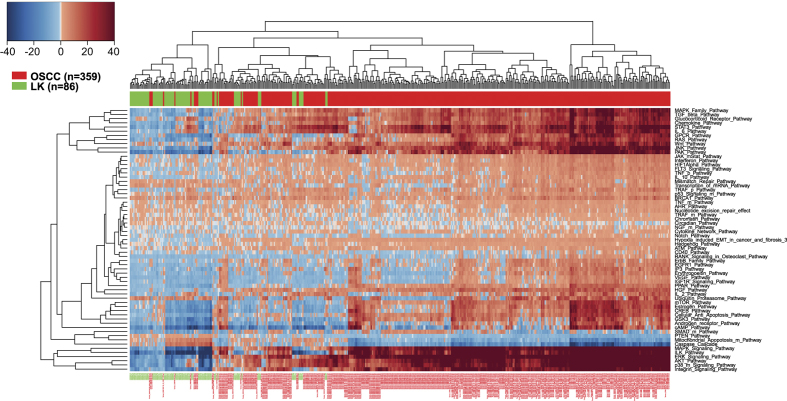
A subset of pre-neoplastic lesions cluster with OSCC at the pathway level. To directly compare pathways activated in OSCC and pre-neoplastic lesions, we have created the hierarchically clustered heatmap of differentially activated pathways in all data sets analyses. Cross-platform normalization and filtering procedures have employed to adjust for the possible batch effect and other variations that may arise during the microarray processing. Red boxes represent pathway upregulation and blue boxes represent pathway downregulation.

To further explore pathways activated in OSCC and leukoplakia, we have created the hierarchically clustered heatmap of differentially activated pathways in all samples analyzed (Figure 2). Although gene expression for OSCC and leukoplakia tissues have been generated using different versions of the Affymetrix profiling platforms, both arrays showed high degree of correspondence.^44^ To adjust for the possible batch effect and other variations that may arise during the microarray processing, we have employed cross-platform normalization and filtering procedures as previously described^45^ (see Materials and Methods section for details). While most of the leukoplakia samples clustered together according to their pathway activation landscape, a subset of 20 pre-neoplastic lesions has clustered toward the center of the heatmap, demonstrating some degree of similarity to the pathway activation profile seen in OSCC (Figure 2, Supplementary Table 10). Similar to OSCC, most of these samples have demonstrated a dramatic upregulation in MAPK, ERK, JNK, IL-6/STAT3, WNT, TGF*β* and glucocorticoid receptor signaling, suggesting that at least in some cases, dysregulation of these signaling networks may be the driving force behind the early stages of OSCC tumorigenesis.

Thirty five of the 86 leukoplakia patients have developed oral cancer over the median of 6.08 years follow-up time post resection of the dysplastic lesion.^22^ Interestingly, only 7 of the 20 leukoplakia patients whose signaling profiles cluster together with tumor signaling profiles have developed an overt neoplasm during the follow-up period. Since benign oral lesions may exist for many years without progression to malignancy, a longer follow-up time may be necessary to further unmask the role of these pathways in early OSCC development. These data also indicate that only a fraction of leukoplakias that eventually progress to malignancy gain aberrations in well-established cancer-driving pathways, suggesting that HNSCC may arise from alterations of different molecular pathways and that there is no single predominant signaling pattern of OSCC progression.

Using the transcriptomic data from the same 86 leukoplakia samples and multiple independent OSCC data sets, Mao and colleagues proposed a 29-trancript prediction model of oral cancer progression (with 8% predicting error rate), indicating that gene expression levels can be effectively used for phenotype prediction.^22^ However, we could not statistically significant stratify patients with leukoplakia according to their progression status based on the pathway activation profiles (not shown), indicating that predisposition for leukoplakia progression may not have significant manifestation on the functional (pathway) level. One possible explanation is that the most differentially expressed genes in a given signature may not be part of the pathways that actually drive tumor behavior. Alternatively, expression of some genes within the cancer-driving pathways is not always predictive of the overall pathway activation. While iPANDA may be a useful tool when used as input for machine learning algorithms to make better prediction models,^34^ we were unable to use modern machine learning methods, since currently available data to system dimensionality ratio precludes the development of a strong classifier at the pathway level. As comprehensive analysis of the tumor pathway activation profile may provide broader clinical utility than raw gene expression evaluation, it would be of paramount importance to reanalyze the data upon accumulation of additional leukoplakia data sets, and further estimate the values of this approach for capturing signaling features that characterize relevant lesions that actually progress to cancers.

## Discussion

An understanding of the protein signaling dynamics during the HNSCC progression is of critical importance for the development of new therapeutic approaches and identification of novel prognostic biomarkers. Signaling pathway activation analysis is a powerful tool for extracting biologically relevant features from large-scale transcriptomic data. While systematic application of such approach may contribute to better understanding of complex signaling networks in various types of cancer, many popular pathway-based methods^29–33^ often fail to provide stable pathway signatures of a specific phenotype. iPANDA, a recently reported method for biologically relevant dimensionality reduction in transcriptomic data, demonstrates better performance for the noise reduction test in comparison to other pathway analysis approaches, and produces highly consistent sets of biologically relevant pathway perturbation signatures on multiple transcriptomic data sets.^34^ Here, we applied this novel bioinformatics prediction algorithm on transcriptomic data from 359 OSCC and 86 oral pre-neoplastic samples.

By utilizing our approach, we were able to detect dysregulation of the major molecular processes involved in HNSCC tumorigenesis (such as those involving PI3K/AKT/mTOR, RAS/RAF/MAPK, JAK/STAT, WNT/*β*-catenin and TGF*β*/SMAD signaling) in almost every OSCC tumor analyzed. Confirming the emerging view that while the specific molecules altered in each individual tumor may be distinct, they all participate in a handful of dysregulated molecular pathways that are likely shared among most HNSCC lesions. Although the association of the above signaling pathways with HNSCC is not novel, the ability of the gene aggregation algorithm to identify highly robust sets of pathway signatures relevant to the HNSCC tumorigenesis, suggests that our platform provides a rational biomathematical framework for studying signaling alterations driving the tumorigenic processes, and further supports the credibility of the pathway activation analysis as a promising analytical approach.^34^ It also suggests that some of the most dysregulated pathway branches identified by our algorithm but not yet wet-lab validated as HNSCC related, may provide an attractive target for future research.

OSCC has been associated with the presence of pre-cancerous dysplastic lesions, thus providing a rational model to elucidate the carcinogenic signaling axes in more detail. In contrast to OSCC, we predicted that survival and mitogenic signaling axes were downregulated and pathways related to apoptosis were slightly upregulated in the majority of leukoplakia samples tested. While it is known that leukoplakia may persist for years without progression to cancer or undergo spontaneous regression or disappearance, there is only limited information on the rate and factors influencing these processes.^46,47^ It is possible, that early lesions demonstrating such signaling phenotype remain stable, unless they acquire oncogenic mutations in pathways shown to be dysregulated in OSCC. On the other hand, since only 7 of the 35 pre-neoplastic lesions resected from patients that eventually developed OSCC have demonstrated tumor-like pathways activation landscape, it is plausible that in some cases pre-malignant lesions and OSCCs are not clonally related to each other, and represent independent neoplastic foci. While the genetic relationship between dysplasia and the associated carcinoma is not yet defined, it is possible that these observations result due to the multifocality of the carcinogenetic process, suggesting a field cancerization state in oral cancer development.^48^

Interestingly, while gene set enrichment (GSEA) analysis of the same microarray data set with GSEA algorithm detected that gene sets related with proteasome machinery, MYC upregulation and ribosomes components were associated with a high risk of oral cancer development,^22^ we were unable to stratify patients with leukoplakia according to their progression status based on our analysis. Although direct comparison of the data generated by different computational pathway scoring platforms should be addressed with caution, GSEA algorithm (and its extensions) relies solely on gene enrichment statistics, treating pathways as unstructured sets of genes.^29^ Whereas our method estimates the topological coefficients for each gene module as a whole, rather than for individual genes inside the module, and therefore outperforms ssGSEA in respect to ranking of the putatively relevant pathways (described in details here).^34^

Although this study focused on fundamental signaling processes involved in the development of OSCC, branched axes radiating from these main tumor-driving pathways may provide safer and more efficient targets for therapeutic intervention than a pan-pathway blockade. Since most of the existing pathways collections are being developed and cataloged within individual research groups, both in industry and in academia, inconsistency in pathway names annotations and number of pathway components (branches) poses a major challenge associated with prediction modeling of subnetworks within a large cancer driver superpathway. Therefore, systematic standardization and optimization of existing signaling pathways databases (such as UniProt, HPRD, QIAGEN SABiosciences, WikiPathways, Ariadne Pathway Studio, SPIKE, Reactome, KEGG and others) will eventually contribute to better resolution of the signaling landscape, and enhance the development of novel prognostic biomarkers for risk assessment and treatment interventions at various stages of cancer progression.

In conclusion, our work poses pathway-based expression data analysis as a promising hypothesis-generating approach for detecting cancer-promoting pathways at different stages of tumorigenesis. However, due to the limited number of currently available leukoplakia data sets, we could not dissect the molecular pathways predictive of progression from pre-neoplasia to carcinoma. As more data sets will become available, transcriptomic analysis of longitudinally collected leukoplakias and tumors that developed in the same patient over time may allow better detection of early drivers of OSCC carcinogenesis on the pathway level.

## Materials and methods

### Source data sets

For this study, we utilized microarray gene expression data obtained from NCBI GEO database. OSCC samples and tissue-specific normal controls used as a reference are listed in Supplementary Table 1 and Supplementary Table 2, respectively. Oral dysplastic cohort and non-tumorous oral mucosa samples profiled on the same platform are summarized in Supplementary Table 5 and Supplementary Table 6, respectively.

### Bioinformatics analysis and transcriptomic expression data pre-processing

OSCC and leukoplakia data with corresponding normal samples were processed using AFFY (version 1.46.1) and FRMA (version 1.20.0) algorithms from Bioconductor (www.bioconductor.org). Microarray raw data were background-adjusted and quantile-normalized using the corresponding R packages (R Core Team, Vienna, Austria). Obtained gene expression values were averaged across all replicates. Heatmap generation and hierarchical clustering were performed using R package gplots. Statistical tests and correlation analysis were done with the MS Excel software (Microsoft Corporation, Redmond, WA, USA).

### Signaling pathway analysis

Pre-processed gene expression data were loaded into pathway analysis software, a proprietary suite developed by Insilico Medicine, Inc. that represents an implementation of the iPANDA algorithm,^34^ optimized for cancer studies. Briefly, the database (collection of signaling pathways most strongly associated with various types of malignant transformation in human cells obtained from the SABiosciences collection (http://www.sabiosciences.com/pathwaycentral.php)) contains a set of 374 signaling pathways that cover a total of 2294 unique genes. Main pathways represent complex signaling networks with multiple downstream targets. Whereas branching axes represent dissected linear signal transduction units of a large multifunctional pathway. Each pathway contains an explicitly defined topology represented as a directed graph. Each node corresponds to a gene or a set of genes, while edges describe biochemical interactions between genes in nodes and/or their products. All interactions are classified as activation or inhibition of downstream nodes. The pathway size ranges from about 20 to over 600 genes in a single pathway. The algorithm accounts for the gene grouping into modules based on the precalculated gene co-expression data (using information from COEXPRESSdb database). Each gene module represents a set of genes that experience significant coordination in their expression levels and/or are regulated by the same expression factors. As described,^37^ it enables calculation of the iPANDA pathway activation scores, a value that serves as a quantitative measure of differential pathway activation between the two states. Pathway scoring is based on the expression level and the role of a particular gene in proprietary maps of 300 of signaling pathways. For estimation of pathway activation, we apply the following rule pathways with positive iPANDA values that are considered upregulated, while negative iPANDA values correspond to downregulated pathways.

### Cross-platform normalization

To perform cross-platform normalization between Affymetrix U133 Plus 2.0 Array and Affymetrix 1.0 ST Array, we utilized XPN algorithm^45^ (R package, CONOR).
